# Endometrial Carcinosarcomas are Almost Exclusively of p53abn Molecular Subtype After Exclusion of Mimics

**DOI:** 10.1097/PGP.0000000000001010

**Published:** 2024-02-02

**Authors:** Jutta Huvila, Amy Jamieson, Jennifer Pors, Lynn Hoang, Jelena Mirkovic, Dawn Cochrane, Jessica N. McAlpine, C. Blake Gilks

**Affiliations:** Department of Pathology, University of Turku, Turku University Hospital, Turku, Finland (J.H.); Department of Gynecology and Obstetrics and Division of Gynecologic Oncology, University of British Columbia, Vancouver, BC, Canada (A.J., J.N.M.); Department of Pathology, BC Cancer, Vancouver, BC, Canada (J.P.); Department of Pathology and Laboratory Medicine, University of British Columbia and Vancouver General Hospital, Vancouver, BC, Canada (L.H., C.B.G.); Department of Pathology, Sunnybrook Health Sciences Centre, Toronto, ON, Canada (J.M.); Department of Molecular Oncology, BC Cancer, Vancouver, BC, Canada (D.C.)

**Keywords:** Endometrial carcinoma, Carcinosarcoma, Molecular subtype, p53abn

## Abstract

Our aim was to assess the molecular subtype(s) and perform a detailed morphologic review of tumors diagnosed as carcinosarcoma in a population-based cohort. Forty-one carcinosarcomas were identified from a cohort of 973 endometrial carcinomas diagnosed in 2016. We assessed immunostaining and sequencing data and undertook expert pathology reviews of these cases as well as all subsequently diagnosed (post-2016) carcinosarcomas of no specific molecular profile (NSMP) molecular subtype (n=3) from our institutions. In the 2016 cohort, 37 of the 41 carcinosarcomas (91.2%) were p53abn, 2 (4.9%) were NSMP, and 1 each (2.4%) were *POLE*mut and mismatch repair deficiency molecular subtypes, respectively. Of the 4 non-p53abn tumors on review, both NSMP tumors were corded and hyalinized (CHEC) pattern endometrioid carcinoma, the mismatch repair deficiency tumor was a grade 1 endometrioid carcinoma with reactive stromal proliferation, and the *POLE*mut tumor was grade 3 endometrioid carcinoma with spindle cell growth, that is, none were confirmed to be carcinosarcoma on review. We found 11 additional cases among the 37 p53abn tumors that were not confirmed to be carcinosarcoma on the review (3 undifferentiated or dedifferentiated carcinomas, 5 carcinomas with CHEC features, 2 carcinomas showing prominent reactive spindle cell stroma, and 1 adenosarcoma). In the review of institutional cases reported as NSMP carcinosarcoma after 2016, 3 were identified (1 adenosarcoma and 2 mesonephric-like adenocarcinoma on review). In this series, all confirmed endometrial carcinosarcomas were p53abn. The finding of any other molecular subtype in a carcinosarcoma warrants pathology review to exclude mimics.

Carcinosarcoma is a high-grade, aggressive endometrial carcinoma histotype that consists of a Mullerian carcinomatous component with an abrupt transition to a malignant metaplastic stromal component. The epithelial component is most commonly serous, high-grade endometrioid, or clear cell but can be of any histologic subtype, and the mesenchymal component can be homologous, that is, contain only cell types native to the uterus or heterologous with extra-uterine cell types such as rhabdomyosarcoma or chondrosarcoma present^1^. Carcinosarcoma accounts for ~4% to 6% of endometrial carcinomas^1–3^. Although uncommon, carcinosarcoma is associated with a very poor prognosis and thus contributes disproportionately to endometrial carcinoma mortality^4^. In recent molecularly classified cohorts, disease recurrence has been described in 32%^5^, 52%,^6^ and 62%^7^ of carcinosarcoma cases and death of disease in 58%^7^ of carcinosarcomas. Confusion in the past about the classification of carcinosarcoma, that is, is it a sarcoma or carcinoma, has resulted in it being understudied, as carcinosarcomas were routinely excluded from study cohorts, including clinical trials.

The majority of carcinosarcomas harbor a *TP53* mutation or have abnormal (mutant pattern) p53 immunostaining in the absence of a pathogenic *polymerase epsilon catalytic subunit (POLE)* mutation and mismatch repair deficiency (MMRd) and thus belong to the p53abn molecular subtype^3,7,8^. There is variation in the distribution of molecular subtype observed in carcinosarcomas in the literature, with p53abn accounting for 50% to 91%, MMRd reported to account for 2.4% to 26.1%, no specific molecular profile (NSMP) for 3% to 19%, and 0 to 10.9% being of *POLE*mut molecular subtype^5–7,9,10^. The aim of this study was to assess the molecular subtype of tumors diagnosed as carcinosarcoma in a population-based cohort and perform a detailed morphologic review with a particular focus on carcinosarcomas of molecular subtypes other than p53abn.

## MATERIALS AND METHODS

### Case Selection

All cases diagnosed as carcinosarcoma based on histopathological examination of a hysterectomy specimen in a national cohort of endometrial carcinomas diagnosed/treated in a single calendar year (2016) from the institutional archives of 29 participating Canadian centers^2,11^ were identified for this study. Clinicopathologic and outcomes data were collected as described previously^2,11^. A second set of cases consisted of all carcinosarcomas diagnosed after 2016 at the authors’ institution that were reported as being of NSMP molecular subtype. This study has been approved by the University of British Columbia institutional research ethics board.

### Histomorphologic Classification and Review

The diagnosis of carcinosarcoma was based on the final diagnosis recorded in the pathology reports^11^, and all these cases were subjected to histopathological review by 2 of the authors (J.H. and C.B.G.). One slide chosen by the participating site as being representative of the tumor was available for review, with all slides reviewed in a subset of cases. For the NSMP carcinosarcoma cases diagnosed after 2016 at the authors’ institutions, all slides were available for review.

### Molecular Subtype Diagnosis

Proactive Molecular Risk Classifier for Endometrial Cancer associated immunomarkers (p53, MMR proteins) and *POLE* mutation testing by targeted next-generation sequencing was done as previously described^2,11^. *POLE*mut assignment was based on a list of 11 agreed-upon pathogenic mutations^12^. Other genes assessed on the next-generation sequencing panel were as described previously and included *TP53*.

Cases with more than 1 molecular feature were classified in accordance with the segregation order and rationale described by León-Castillo et al^13^.

## RESULTS

**FIG. 1:**
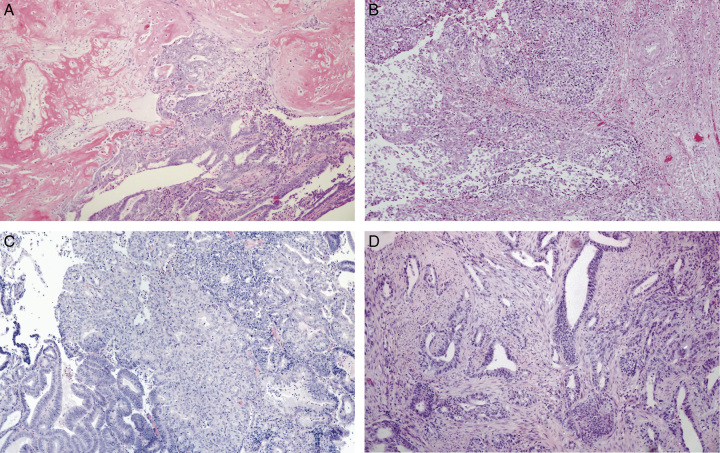
Four cases originally diagnosed as carcinosarcoma that was not p53abn molecular subtype: (A) NSMP tumor, on pathology review diagnosed as corded and hyalinized endometrioid carcinoma (CHEC); (B) Second NSMP tumor, also CHEC rather than carcinosarcoma; (C) POLEmut tumor, grade 3 endometrioid carcinoma with spindle cell growth but without bona fide sarcomatous growth; (D) MMRd tumor, grade 1 endometrioid carcinoma with reactive stromal proliferation. MMRd indicates mismatch repair deficiency; NSMP, no specific molecular profile.

**FIG. 2:**
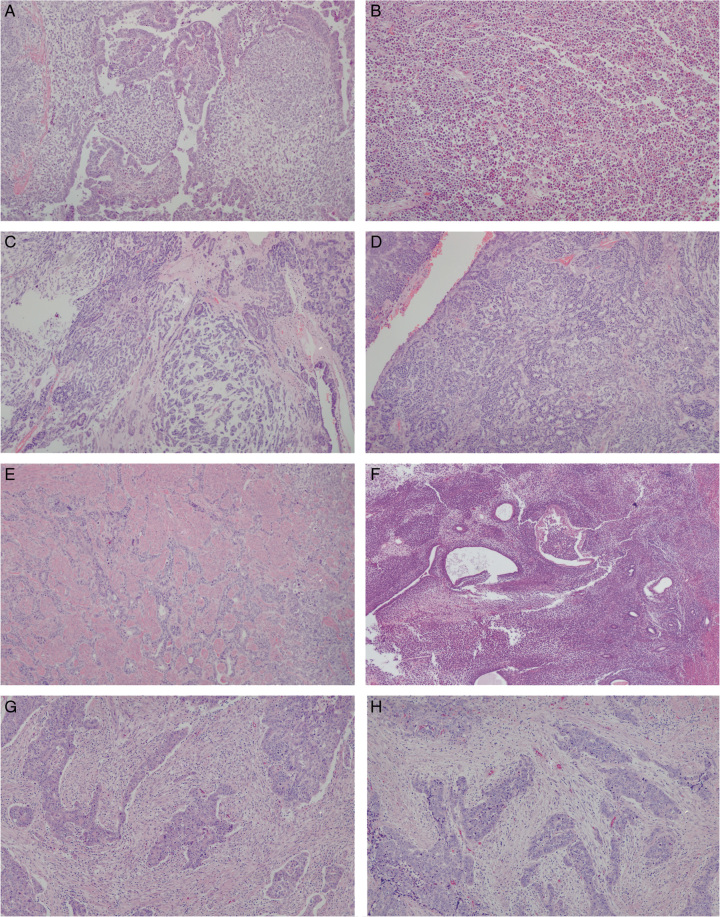
Tumors of p53abn molecular subtype originally diagnosed as carcinosarcoma, where the diagnosis was revised, as follows, based on pathology review: (A) Dedifferentiated carcinoma; (B) Undifferentiated carcinoma; (C) Corded and hyalinized endometrioid carcinoma (CHEC); (D) CHEC; (E) CHEC; (F) Adenosarcoma; (G) Reactive spindle cell stroma; (H) Reactive spindle cell stroma. In none of these tumors was sarcomatous growth identified.

There were 41 carcinosarcomas among the 973 hysterectomy specimens (4.2%) in the cohort of cases from 2016; 37 (91.2%) were p53abn, 2 (4.9%) were NSMP, and 1 each (2.4%) were *POLE*mut and MMRd, respectively. All 37 p53abn carcinosarcomas (as diagnosed based on the presence of mutant pattern p53 immunostaining together with wild-type *POLE* and expression of MMR proteins, that is, MMR proficient) had a *TP53* mutation confirmed by sequencing. Of the 4 non-p53abn tumors (Fig. 1), on review both NSMP tumors were corded and hyalinized (CHEC) pattern endometrioid EC (EEC). The MMRd tumor was a grade 1 EEC with reactive stromal proliferation, and the *POLE*mut tumor was grade 3 EEC with spindle cell growth, that is, none were confirmed to be carcinosarcoma. Eleven additional cases among the 37 p53abn tumors were not confirmed to be carcinosarcoma on review; 3 undifferentiated or dedifferentiated morphology, 5 endometrioid carcinomas with CHEC features, 2 carcinomas with prominent reactive spindle cell stroma rather than a sarcomatous component, and 1 adenosarcoma (Fig. 2 – representative images).

**FIG. 3:**
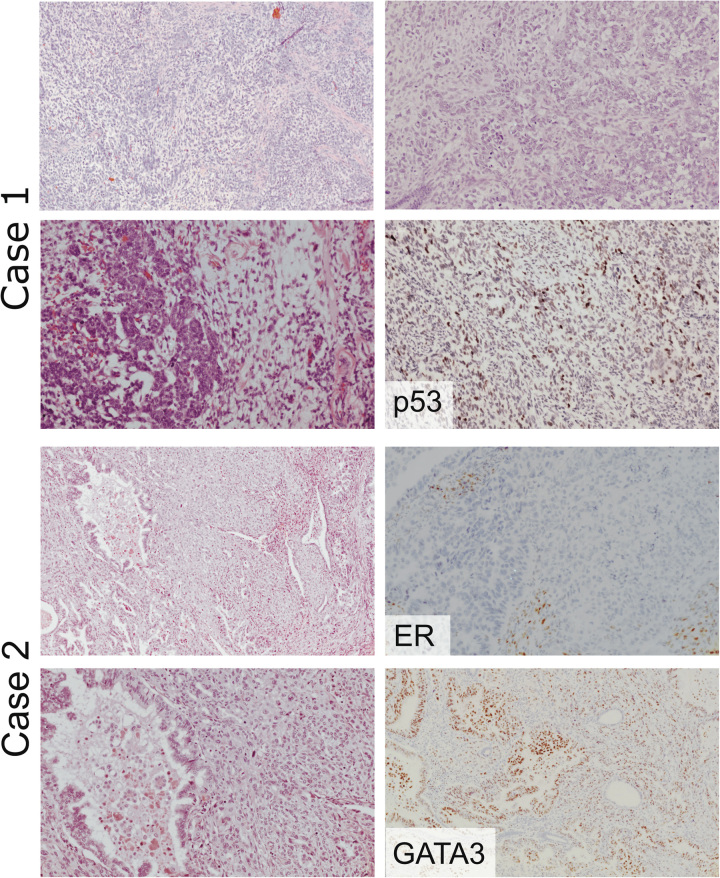
Two cases were originally diagnosed as carcinosarcoma, both of NSMP molecular subtype, which at review were diagnosed as mesonephric-like adenocarcinoma. Case 1: H&E stainings and wild-type p53 staining; Case 2: H&E stainings, negative ER in tumor cells (stromal cells ER-positive) and positivity in GATA3 staining. ER indicates estrogen receptor; GATA3 GATA Binding protein 3; H&E, hematoxylin-eosin stain; NSMP no specific molecular profile.

In the review of institutional cases diagnosed as NSMP carcinosarcoma after 2016, 3 cases were identified. On pathology review, one was reclassified as an adenosarcoma, and 2 as mesonephric-like adenocarcinoma with spindled cells grown and without heterologous elements. (Fig. 3 – mesonephric-like cases).

## DISCUSSION

We show that all pathologically confirmed endometrial carcinosarcomas in a multicenter study are p53abn molecular subtypes. In the literature, the distribution of molecular subtypes of carcinosarcomas is more variable; p53abn account for 50% to 91%, MMRd for 2.4% to 26.1%, NSMP for 3% to 19%, and the *POLE*mut from 0% to 10.9%^5–7,9^ (summarized in Table 1). There are a number of possible explanations for this variability. One significant problem identified in this study, however, was tumors originally diagnosed as carcinosarcoma that, based on diagnostic criteria and practice in 2023, would be diagnosed as something different. We identified diagnostic difficulties with known mimics of carcinosarcoma, such as CHEC pattern endometrioid carcinoma^14,15^, dedifferentiated/undifferentiated carcinoma^16,17^, and mesonephric-like carcinoma with spindle cell growth^15,18,19^. All of these are recently described entities, and although they are included in the fifth edition of the WHO Classification of Female Genital Tumors^1^, awareness of the range of appearances and diagnostic criteria for the distinction of these tumor types from carcinosarcoma remains work in progress. The subsequent institutional follow-up series of cases seen after 2016 identified 3 NSMP carcinosarcomas, of which 2 on review were mesonephric-like adenocarcinomas. These more recent cases add further evidence that carcinosarcomas of molecular subtypes other than p53abn are rare. Thus, although significant numbers of carcinosarcomas have been reported to be molecular subtypes other than p53abn (Table 1), we believe that our findings demonstrate that almost all carcinosarcomas, as diagnosed based on current diagnostic criteria^1^, are p53abn. This has significance in practice, as carcinosarcoma is an “aggressive histologic type” according to the FIGO 2023 staging of endometrial cancer, and p53abn is an aggressive molecular subtype^20^; as such, a diagnosis of either carcinosarcoma or p53abn can result in a change of tumor stage (upstaging). Based on our results, though, the molecular subtype of carcinosarcomas will consistently be p53abn, and situations where the stage of a given tumor will differ based on histotype (carcinosarcoma) versus molecular subtype will be rare.

**TABLE 1:** Molecular Subtypes of Carcinosarcoma^5–7,9,10^

		n (%)			
	n (total)	POLE	MMRd	NSMP	p53abn
Le Gallo et al^9^	53	2 (3.8)	3 (5.7)	10 (18.9)	38 (71.7)
McConechy et al^6^	30	0 (0.0)	1 (3.3)	2 (6.7)	27 (90.0)
Cherniack et al^7^	57	1 (1.8)	2 (3.5)	2 (3.5)	52 (91.2)
Gotoh et al^5^	92	10 (10.9)	24 (26.1)	9 (9.8)	49 (53.3)
Rios-Doria et al^10^	223	0 (0.0)	14 (6.3)	14 (6.3)	195 (87.4)
	455	13 (2.9)	44 (9.7)	37 (8.1)	361 (79.3)

MMRd indicates mismatch repair deficiency; NSMP indicates no specific molecular profile.

It is well established that most MMRd and *POLE*mut endometrial carcinomas are of endometrioid histotype, although *POLE*mut endometrial carcinomas have been known to be difficult to histotype reproducibly due to significant intratumoral heterogeneity of the tumor morphology^21,22^. *POLE*mut carcinosarcomas have been described in the literature, but all reports were published before the definition of pathogenic *POLE* mutations in early 2020 by Leon-Castillo et al^12^. In a recent *POLE*mut individual patient meta-analysis of the literature, restricted to only those tumors with pathogenic *POLE* mutations, there were no carcinosarcomas among the 297 cases^23^. Our carcinosarcoma cohort included 1 MMRd and 1 *POLE*mut tumor, of which the former showed reactive stromal proliferation and the latter spindled cell growth, and neither was confirmed to be a carcinosarcoma on pathologic review. A carcinosarcoma that is MMRd or *POLE*mut would create challenges in treatment planning (Should treatment be based on histotype or molecular subtype?). Gotoh et al showed that *POLE*mut and MSI-high carcinosarcomas had more favorable outcomes compared with NSMP carcinosarcomas, which had a similar prognosis to p53abn^5^. Similar findings were also reported in a subsequent meta-analysis^24^, but the diagnoses of both carcinosarcoma and molecular subtypes are not consistently based on current diagnostic criteria for these previous studies.

In undertaking this study, 1 question we wished to address was whether NSMP carcinosarcoma existed, and if so, was the prognosis similar to or better than that of the more common p53abn carcinosarcoma. We identified no such cases, allowing us to conclude that NSMP carcinosarcoma will be encountered infrequently in practice and making studies of NSMP carcinosarcoma technically challenging or impossible because of their rarity. If such a case were encountered, it would be classified as an aggressive endometrial carcinoma histotype according to FIGO 2023 and treated as such according to the European Society of Gynaecological Oncology-The European SocieTy for Radiotherapy & Oncology- The European Society of Pathology and European Society of Medical Oncology guidelines, with adjuvant chemotherapy +/- radiation recommended in most cases; the absence of data notwithstanding, this approach seems reasonable.

An unexpected finding in this study was the number of cases from the 2016 cohort where the diagnosis of carcinosarcoma was changed on pathology review. Although the focus of this study was not on diagnostic criteria and differential diagnosis, several observations can be made based on our reviews. In the past, it was possible to diagnose as carcinosarcoma any high-grade carcinoma with biphasic growth that included areas of spindle cell growth or loss of overt epithelial differentiation. There is now an emphasis on the presence of bona fide sarcomatous growth, with the cytologically high-grade epithelial and sarcomatous components “sharply juxtaposed,” that is, an abrupt transition between the components^1^, a significant refinement of the diagnostic criteria. CHEC pattern endometrioid carcinomas can be high-grade and often have osteoid or chondroid matrix^25^ but should lack either overt sarcomatous differentiation or this abrupt transition. Dedifferentiated carcinoma is only recently characterized, and many or most such tumors would have been diagnosed as carcinosarcoma in the past based on their biphasic growth, but the undifferentiated component consists of more uniform cells without the pleomorphism and spindle cell morphology of the sarcomatous component of carcinosarcoma^26^. Reactive spindle cell stroma has perhaps been underappreciated as a mimic of carcinosarcoma. As almost all carcinosarcomas show mutant pattern p53 staining, the absence of such staining in the spindle cell area, as well as the more uniform nuclear features of the spindle cells compared with true sarcomatous growth, can be used to support a diagnosis of reactive stroma rather than carcinosarcoma.

Does the distinction between these other diagnostic entities and carcinosarcoma matter in a tumor that is p53abn, that is, is it clinically relevant? The larger question becomes whether there are meaningful subcategories within p53abn endometrial carcinoma that can be recognized based on histopathological features. In 1 meta-analysis, p53abn carcinosarcomas had an adverse progression-free survival compared with p53abn endometrioid and serous carcinomas^24^; however, in recent studies, no difference in progression-free or overall survival was found between serous carcinoma, endometrioid carcinoma and carcinosarcoma of p53abn molecular subtype^10,27^ providing evidence of histotype being prognostically irrelevant in the context of p53abn molecular subtype, when the latter is known. Although there are opportunities for future studies of p53abn endometrial carcinoma to determine whether morphologic variants such as endometrioid carcinoma with CHEC pattern are of prognostic significance within this high-risk molecular subtype, it will be important that such studies address not only prognosis but provide reliable diagnostic criteria that allow for reproducible diagnosis; histotype diagnoses for high-grade endometrial carcinomas have historically suffered from suboptimal reproducibility^28,29^. It is telling that the ESGO/ESTRO/ECP, European Society of Medical Oncology, and FIGO 2023 risk assessment guidelines/staging guideline base risk category (and thus treatment) primarily on molecular subtype rather than histotype when molecular subtype is known^20,30,31^.

CHEC pattern endometrioid carcinoma is often low-grade and associated with loss of MMR proteins or of NSMP subtype, although high-grade CHEC pattern tumors associated with p53 abnormalities have been described^14,32^. CHEC pattern is rare and if associated with low-grade carcinoma and in the absence of mutant pattern, p53 immunostaining is associated with a favorable prognosis^33,34^, so separation from carcinosarcoma is important in that scenario. If, however, the carcinoma is high-grade and/or there is a mutant pattern p53 immunostaining in a CHEC pattern endometrioid carcinoma, such a distinction may not be clinically relevant, as noted above. Dedifferentiated/undifferentiated carcinoma is a very aggressive histotype of endometrial carcinoma, but the molecular abnormalities most commonly are mutations in genes encoding proteins in the SWI/SNF chromatin remodeling complex and frequent MLH1 promoter methylation, with wild-type *TP53*. The underlying molecular pathology of dedifferentiated/undifferentiated carcinoma does not impact treatment at this time, but there are research efforts directed at developing more targeted therapies against cells with mutations in the SWI/SNF chromatin remodeling genes,^35^ and correct identification of these tumors may determine treatment in the future. Mesonephric-like carcinomas are mostly of NSMP molecular subtype^19,36^; the diagnostic histopathological features and molecular pathology are being elucidated, but this is an important subset of endometrial carcinoma even though uncommon, as it is typically architecturally low-grade but is associated with aggressive behavior. In a recent study, Mirkovic et al reported observations on mesonephric-like carcinosarcomas and recommended that a diagnosis of mesonephric-like carcinosarcoma should be reserved for neoplasms with heterologous mesenchymal elements as mesonephric-like carcinomas often have spindle cell elements. As with the dedifferentiated/undifferentiated carcinomas, the diagnosis of mesonephric-like adenocarcinoma/carcinosarcoma does not result in specific treatment differences at this time, but accurate diagnosis of this histotype is an important starting point in the process of developing more personalized treatments based on the molecular pathology.

Disagreement in the past about the classification of carcinosarcomas, that is, sarcoma versus carcinoma, has resulted in it being significantly understudied, and carcinosarcomas were routinely excluded from study cohorts, including clinical trials. A recent endometrial carcinosarcoma consensus statement has put a call to action to design ad hoc endometrial carcinosarcoma-oriented studies to develop new practice changing targeted therapies and to generate data to provide specific guidelines for the management of endometrial carcinosarcoma^37^. Accurate diagnosis of carcinosarcoma to generate this data will be crucial. Our study identified several diagnostic difficulties with known mimics of carcinosarcoma in modern pathology practice, as the diagnosis of “carcinosarcoma” has evolved from a purely descriptive diagnosis to being based on specific criteria (a trend that has happened with a number of gynecologic cancers, such as clear cell carcinoma or small cell carcinoma, where the name reflects a prominent histologic feature that led to them being identified as a distinct tumor type, but the current diagnostic criteria incorporate other features, including immunohistochemical or other molecular pathology markers, and are not based solely on the notable feature after which they are named). More than a third of the carcinosarcomas diagnosed in 2016 were reclassified as other histotypes when following the 2020 WHO fifth edition diagnostic criteria. We recommend that all non-p53abn carcinosarcomas undergo expert gynecopathologic review before being enrolled in a clinical trial, and that special attention be paid to mimics of carcinosarcoma such as dedifferentiated/undifferentiated carcinoma, CHEC pattern endometrioid carcinoma, mesonephric-like carcinosarcoma/carcinosarcoma, and carcinoma with reactive spindle cell stroma.
